# Predominant negative symptoms in female schizophrenia in-patients: An observational study

**DOI:** 10.1192/j.eurpsy.2025.564

**Published:** 2025-08-26

**Authors:** J. Dragasek, Z. B. Dombi, P. Herman, V. Dzurilla, Á. Barabássy

**Affiliations:** 1 University of P. J. Safarik, Kosice, Slovakia; 2 Gedeon Richter Plc., Budapest, Hungary; 3 Gedeon Richter Slovakia, Bratislava, Slovakia

## Abstract

**Introduction:**

Negative symptoms are a key aspect of schizophrenia, significantly impacting a patient’s functioning and quality of life. Although hospitalizations are often associated with positive symptoms, negative symptoms can also dominate the clinical picture in in-patients. Female patients are usually underrepresented in schizophrenia studies.

**Objectives:**

To analyse the changes of negative symptoms in female in-patients.

**Methods:**

This was an observational study with data recorded at hospital entry and release. Adult inpatients with a schizophrenia diagnosis according to the International Classification of Diseases 10th edition who exhibited predominant negative symptoms according to clinical judgement were included. Patients received pharmacological and some non-pharmacological treatment as usual.

The primary outcome measure was the modified Short Assessment of Negative Domains (m-SAND), an anamnesis-based scale that is composed of 7 items: two positive items (delusions and hallucinations) and five negative items (anhedonia, alogia, avolition, asociality and affective flattening). Each item is rated from 0 to 5 (not observed; mild; moderate; moderately severe; severe; and extreme). Other measurements included the Self-evaluation of Negative Symptoms (SNS).

Least squares (LS) means were calculated for the change from baseline to final visit using a mixed model for repeated measures (MMRM).

**Results:**

63 female patients were included in the study. The mean age was 41.6 years with 14.2 years of mean duration of illness. All patients had predominant negative symptoms, in fact, 65% of them was hospitalized because of it. 30% of the patients also had secondary negative symptoms, mainly due to positive symptoms. The mean duration of hospital stay was 38 days. All patients received pharmacotherapy. At baseline, 9.5% were on cariprazine monotherapy and 84.1% on cariprazine combined with another antipsychotic such as olanzapine (23.8%) or quetiapine (7.9%). By the end of the hospital stay, 14.3% of female patients received cariprazine monotherapy and 82.5% cariprazine combination treatment with olanzapine (30.2%) or clozapine (15.9%). Significant decrease was detected in m-SNAD total score (LS mean change from baseline: -10.95) and SNS total score (LS mean change from baseline: -9.74). Functioning increased from poor (76%) to ‘manifest disabilities’ according to PSP (81%).

**Conclusions:**

In summary, female patients had significant improvement during their hospital stay in terms of negative symptoms. The most utilized pharmacotherapy during the hospital stay was cariprazine both in a form of mono- and polytherapy.

**Disclosure of Interest:**

J. Dragasek: None Declared, Z. Dombi Employee of: I am an employee of Gedeon Richter Plc., originator of cariprazine., P. Herman Employee of: I am an employee of Gedeon Richter Plc., originator of cariprazine., V. Dzurilla Employee of: I am an employee of Gedeon Richter Plc., originator of cariprazine., Á. Barabássy Employee of: I am an employee of Gedeon Richter Plc., originator of cariprazine.

